# Correlation Between the Prognosis of Sudden Total Deafness and the Peripheral Blood Inflammation Markers

**DOI:** 10.3389/fneur.2022.927235

**Published:** 2022-06-15

**Authors:** Tongxiang Diao, Yujie Ke, Junbo Zhang, Yuanyuan Jing, Xin Ma

**Affiliations:** ^1^Department of Otolaryngology, Head and Neck Surgery, People's Hospital, Peking University, Beijing, China; ^2^Department of Otolaryngology, Head and Neck Surgery, Peking University First Hospital, Beijing, China

**Keywords:** peripheral blood inflammation markers, prognosis, sudden total deafness, PLR, inflammation

## Abstract

**Objective:**

To analyze the correlation between prognosis of sudden total deafness (STD) and peripheral blood inflammation markers including white blood cell count (WBC), monocytes, neutrophil/lymphocyte ratio (NLR), platelet/lymphocyte ratio (PLR), fibrinogen (FIB).

**Methods:**

125 patients with STD who were hospitalized in our department from 2014 to 2019 were enrolled. The general physical conditions, clinical manifestations, pure tone audiometry, imaging examination, and peripheral blood inflammation markers were collected, and all patients were divided into effective and ineffective two groups according to the degree of hearing recovery at the time of discharge. Then binary logistic regression was used to analyze the correlation between multiple factors and prognosis, meanwhile the receiver operating characteristic (ROC) curve was used to evaluate the predictive value of the above prognostic factors.

**Results:**

Compared with the ineffective group, patients in the effective group were younger and have higher PLR level and lower FIB levels. Age and PLR are independent prognostic factors. Taking age ≤ 56 years old, PLR >142.6 as the standard to predict the prognosis of patients with STD has the largest AUC with the potential effective rate reaching 78.1%.

**Conclusions:**

Age and PLR are independent prognostic factors for patients with STD. The younger the age and the higher the PLR, the better the prognosis. Clinically, the prognosis of patients with STD can be evaluated by the patient's age and PLR level, which is of great significance to predict the prognosis of patients with STD.

## Introduction

Sudden sensorineural hearing loss (SSHL) refers to sensorineural hearing loss with no identifiable cause that occurs within 72 h. The Clinical Practice Guideline: Sudden Hearing Loss of American Academy of Otolaryngology (2019) defines it as a decrease in hearing of ≥30 dB affecting at least 3 consecutive frequencies, may be accompanied by tinnitus, ear fullness, numbness, dizziness and other discomforts. Clinically, it usually manifests as unilateral disease, and bilateral SSHL is rare. According to previous studies, the incidence of SSHL is 5-160/100,000 (1, 2), and there is an increasing trend year by year. The etiology and pathophysiological mechanism of sudden deafness have not been fully elucidated. At present, the mechanisms that may be related include blood vessels, immunity, viruses, inner ear hydrops, etc. In recent years, with the hypothesis of pathological activation of cellular stress pathways, chronic inflammation has drawn more attention for its important role in the pathogenesis of SSHL.

Chronic inflammation has been proved to be an important risk factor for microvascular injury and atherosclerosis (3). Researchers believe that it can also damage the vascular endothelial function of the inner ear, leading to hearing loss (4). The correlation between peripheral blood inflammatory markers, such as white blood cell (WBC) count, WBC subtype count, neutrophil to lymphocyte ratio (NLR) and platelet to lymphocyte ratio (PLR) and SSHL has been widely recognized by clinical researchers. At present, it is considered that these inflammatory markers, especially NLR, are significantly correlated with the SSHL (5, 6), However, the correlation between NLR and PLR and the prognosis of SSHL is still controversial (6, 7). SSHL has great heterogeneity. The degree of hearing loss, audiogram shapes and different accompanying symptoms may suggest different pathologies. Previous studies have not classified SSHL, which may explain the inconsistent conclusions. According to Chinese guidelines for the diagnosis and treatment of sudden deafness (2015) (8), sudden total deafness (STD) refers to absent response at all tested frequencies, with an average hearing threshold ≥80 dB HL (2015) (8). Because of the severe hearing loss, patients with STD always be accompanied with many symptoms, including tinnitus (9), dizziness or vertigo (10), numbness around the ear (11), anxiety, depression, and insomnia, which would greatly reduce the quality of life. This study chose patients with STD as the research subjects to explore the relationship between the peripheral blood inflammatory markers and the prognosis of STD, trying to seek for markers that may indicate the prognosis of STD.

## Materials and Methods

### Study Subjects

Between Jan 2014 and Jan 2019, eligible patients with STD who were hospitalized in our department for treatments were continuously enrolled. The detailed inclusion criteria were: (1) sudden sensorineural hearing loss occurred within 72 h; (2) the hearing thresholds of all frequencies were increased and were nearly consistent, with an average hearing threshold of 250–8,000Hz (250, 500, 1,000, 2,000, 3,000, 4,000, and 8,000 Hz) ≥80dBnHL; (3) the time from onset of hearing loss to treatments was <30 days. (4) receive no treatment before admission. (5) acoustic neuroma and other diseases are excluded from CT or MRI. Patients who could not cooperate due to severe mental factors and patients whose hearing loss had clear causes were excluded, such as excessive noise exposure, Meniere's disease, middle ear structure malformation, post-cochlear diseases, and histories of ototoxic drug use, head trauma, and ear surgery.

A total of 125 patients with STD were finally included. All pure-tone audiometry tests were performed by a same audiologist in a soundproof room using the Interacoustics Clinical Audiometer AC40. The following baseline information of these patients were collected for analysis, including sex, age, affected ear side, body mass index (BMI), disease course (the time from onset to therapy), accompanying symptoms such as tinnitus or dizziness, the average value of 250, 500-, 1,000-, 2,000-, 3,000, 4,000, and 8,000-Hz air conduction pure tone average hearing thresholds (PTA) of affected ear, chronic diseases history such as hypertension, diabetes, and hyperlipidemia. The blood routine data included white blood cell count (WBC), neutrophil count (NEU), monocyte count (MONO), platelet count (PLT), neutrophil/lymphocyte count ratio (NLR), platelet/lymphocyte count ratio (PLR), and fibrinogen level (FIB).

### Treatments

The treatments of this group of patients were nearly the same according to Chinese guidelines for treating sudden deafness in 2015 (8), which included ginkgo biloba extract injection for improving microcirculation, batroxobin for decreasing plasma fibrinogen level, mecobalamin, glucocorticoid, etc.

### Evaluation of Treatment Efficacy

The treatment efficacy was evaluated by comparing hearing thresholds of impaired frequencies (IF) before and 2–4 weeks after treatments. A patient was defined as effective if the IF of affected ear decreased by 15 or more than 15 dB. While when the IF of affected ear decreased by <15 dB a patient was defined as in-effective (2015).

### Ethics Statement

The Peking University People's Hospital Ethical permission committee approved study (2021PHB149) and all subjects provided written informed consents.

### Statistical Analysis

All the statistical analysis of this study were completed by SPSS 20.0 soft package (IBM, Armonk, NY, USA). All continuous data were displayed as mean±standard deviation. The unpaired student's *t*-test was used to compare the quantitative variables among different groups. Pearson's Chi-square test was used to compare the categorical variables among different groups. Multiple logistic regression analysis was used to evaluate the significance of independent variables of treatment efficacy. The Receiver Operating Characteristic (ROC) curve-test was used to determine the best cutoff value of independent variables. A *p* < 0.05 was considered to be statistically significant.

## Results

### Baseline Information

Totally, among the 125 patients with STD 68 patients were effective and 57 patients were in-effective, with an overall effective rate of 54.4%. The comparisons of baseline information before treatments were shown in Table 1, suggested significantly smaller age, higher PLR value, and lower FIB value in effective group than in in-effective group (all *P* < 0.05). No other factors differed significantly between these two groups of patients (all *P* > 0.05).

**Table 1:** The comparisons of baseline information between effective and in-effective group.

	**All patients (*n =* 125)**	**Effective group (*n =* 68)**	**In-effective group (*n =* 57)**	**P**
Age (years)	50.0 ± 17.4	46.6 ± 17.5	54.1 ± 16.5	0.015*
BMI (kg/m^2^)	24.5 ± 3.2	24.4 ± 3.3	24.7 ± 3.0	0.632
Affected side (left)	62	34	28	1.000
Sex (female)	63	32	31	0.474
Onset-therapy (days)	10.38 ± 9.44	9.59 ± 8.81	11.32 ± 10.15	0.310
PTA (dB)	106.9 ± 14.1	107.3 ± 13.7	106.3 ± 14.5	0.694
WBC	8.5 ± 3.0	8.7 ± 3.2	8.3 ± 2.8	0.432
NEU	6.1 ± 2.9	6.4 ± 3.1	5.8 ± 2.6	0.330
NLR	3.9 ± 2.7	4.3 ± 2.9	3.5 ± 2.4	0.131
PIT	231.4 ± 60.1	240.9 ± 62.0	220.1 ± 56.2	0.051
PLR	146.2 ± 8.0	160.1 ± 94.3	129.8 ± 56.2	0.028*
MONO	0.4 ± 0.2	0.4 ± 0.2	0.4 ± 0.2	0.979
FIB	216.3 ± 105.0	197.6 ± 100.8	238.6 ± 106.4	0.030*
Dizziness (Yes)	68	34	34	0.367
Tinnitus (Yes)	105	61	47	0.298
Diabetes (Yes)	21	11	10	1.000
Hypertension (Yes)	35	21	14	0.549
Hyperlipemia (Yes)	15	7	8	0.585

*BMI, body mass index; PTA, pure tone average hearing thresholds; WBC, white blood cell count; NEU, neutrophil count; NLR, neutrophil/lymphocyte count ratio; PIT, platelet count; PLR, platelet/lymphocyte count ratio; MONO, monocyte count; FIB, fibrinogen level*.

*P < 0.05.

### Independent Predictors for Prognosis of Patients With Sudden Total Deafness

In order to further explore independent predictors for prognosis of patients with STD, the pre-treatment factors which differed significantly between effective and in-effective group, including age, PLR value, and FIB value, were all incorporated into a logistic analysis for predicting treatment response. As shown in Table 2, only age and PLR value could be included, and the OR values were 1.024 for age (95%CI: 1.002–1.048) and 0.993 for PLR (95%CI: 0.988–0.999), respectively.

**Table 2:** Logistic predictors to prognosis of patients with STD.

	**OR value**	**95%CI**	***P***
Age (years)	1.024	1.002–1.048	0.036*
PLR (10^9^/L)	0.993	0.988–0.999	0.022*
FIB	1.004	1.000–1.007	0.063

*STD, sudden total deafness; PLR, platelet/lymphocyte count ratio; FIB, fibrinogen level*.

*P < 0.05.

### Predictive Efficacies of Age and PLR for Prognosis of Patients With Sudden Total Deafness

**Figure 1:**
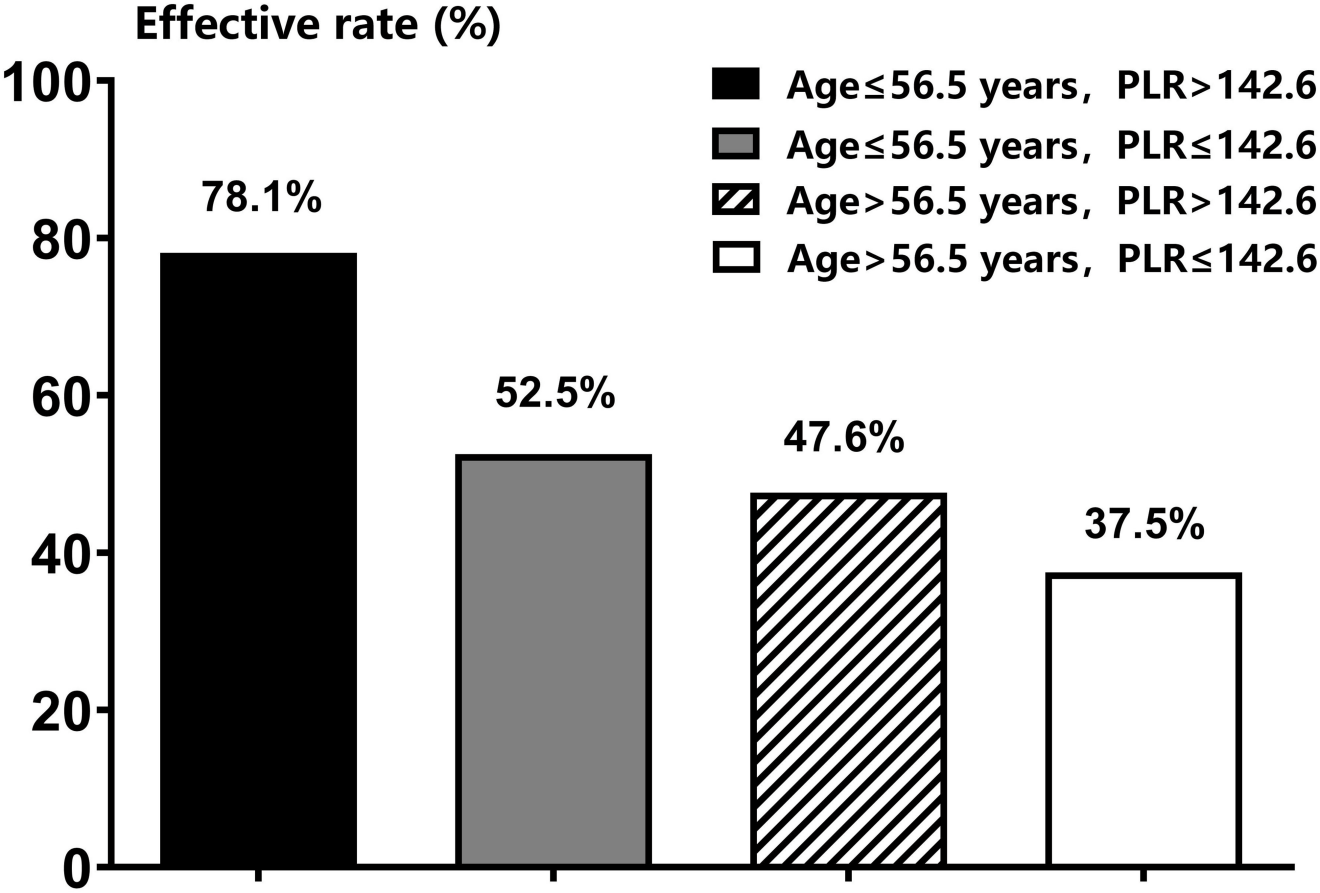
The effective rates between sudden total deafness patients with different age and PLR values.

The predictive efficacies for prognosis of patients with STD were all analyzed by ROC curve-test. As shown in Table 3, the area under the curve (AUC) calculated was 0.660 for the combination of age and PLR value, which was higher than that of both age alone (0.628) and PLR alone (0.589), suggested a higher predictive efficacy of the combination of these two factors. According to Yorden Index, the best cut-off value for age was 56.5 years with sensitivity and specificity of 54.4 and 67.6%, respectively, and the best cut-off value for PLR was 142.6 with sensitivity and specificity of 51.5 and 68.4%, respectively. As shown in Table 4 and Figure 1, the effective rates of different groups of STD patients according to such cut-off values were significantly different (*P* < 0.05). The effective rate of patients with age of ≤ 56 years and PLR value of >142.6 could reach to 78.1%, which was significantly higher than that of all the other three groups of patients (*P* < 0.05).

**Table 3:** The predictive efficacies of age, PLR value, and the combination of age and PLR value for prognosis in patients with STD.

	**AUC**	**95%CI**	**Best cut-off value**	**Sensitivity**	**Specificity**	***P***
Age	0.628	0.530~0.726	56.6	54.4%	67.6%	0.014
PLR	0.589	0.489~0.688	142.6	51.5%	68.4%	0.088
Combination	0.660	0.565~0.754	–	64.9%	60.3%	0.002

*STD, sudden total deafness; PLR, platelet/lymphocyte count ratio*.

**Table 4:** The effective rates according to different age and PLR values.

	**Effective rate**	***P***
Age ≤ 56.5 years, PLR>142.6	78.1% (25/32)	0.010*
Age ≤ 56.5 years, PLR ≤ 142.6	52.5% (21/40)	
Age > 56.5 years, PLR>142.6	47.6% (10/21)	
Age > 56.5 years, PLR ≤ 142.6	37.5% (12/32)	

*PLR, platelet/lymphocyte count ratio.*

*P < 0.05.

## Discussion

This study found that age was an independent prognostic factor of sudden total deafness. The younger the age, the better the prognosis, which is consistent with previous studies (12). Previous studies have shown that sudden deafness can occur at all ages, mainly in adults aged 18–59. Overwork and emotional fluctuation are the main inducing factors. This may be due to certain degenerative changes occurs in vascular wall compliance, microcirculation hemorheology and inner ear auditory function with age.

In recent years, with the draw of pathological activation of cell stress pathway hypothesis, people began to realize that inflammatory response may play an important role in the pathogenesis of sudden deafness. At present, the research on the etiology of SSHL mainly focuses on its relation with chronic inflammation (13). It is believed that the inner ear is a “terminal organ” without collateral circulation, and its blood supply mainly depends on the labyrinth artery. The hair cells in the cochlea consume a lot of oxygen and are extremely sensitive to hypoxia. Therefore, the reduction of blood flow will lead to SSHL. In addition, due to the clinical characteristics of acute onset and unilateral multiple, SSHL is considered to be similar to the pathogenesis of ischemic diseases such as myocardial infarction, transient ischemic attack or transient amaurosis (14). So we deemed that in addition to the inner ear circulation disorder, chronic inflammation also plays an important role in the occurrence of SSHL. The inflammatory reaction could destroy the vascular endothelium and damage the blood flow of the inner ear, trigger the formation of atherosclerosis, then damage the vascular stria and increase the risk of ischemia (6). The wide application of anti-inflammatory steroids in the treatment of SSHL also confirms the important role of inflammation in the pathogenesis.

The peripheral blood inflammatory cells mainly include leukocytes, neutrophils, lymphocytes and platelets, which play important roles in the control of inflammation. These inflammatory markers and the ratios obtained from these markers, such as NLR and PLR, have been concerned by many researchers as markers of systemic inflammation (15, 16), for they are easy to collect, cheap, and has the same predictive effect with those expensive markers, such as interleukin (IL)-6, IL-8, IL-1β, and tumor necrosis factor- α (17). The correlation between inflammatory markers, especially NLR, PLR and sudden deafness has been widely recognized by clinical researchers. However, there is still controversy about their correlation with the prognosis of sudden deafness. Chen et al. (6) found that NLR and PLR, as comprehensive indicators of two complementary immune pathways, have a certain correlation with the occurrence and prognosis of SSHL, namely NLR and PLR can be used as biomarkers for the diagnosis and prognosis of SSHL. Cao et al. (16) also reported similar correlation between PLR, NLR and SSHL in their meta-analysis. Seo et al. found that the levels of NLR and PLR in patients with SSHL were significantly higher than those of control group, while the level of NLR in the recovered group was lower than the un-recovered group (18). Durmuş et al. in 2016 also reported that the NLR and PLR values of SSHL patients were higher than the control group, and un-recovered SSHL patients had higher NLR and PLR levels than the recovered-group (17). Therefore, it is considered that NLR and PLR are of certain value in predicting the prognosis of SSHL. Similarly, Kum et al. (19) also reported higher NLR levels in un-recovered SSHL patients, but the average platelet volume, a sensitive indicator of platelet activity, was not significantly correlated with the prognosis. Ikinciogullari et al. (20) also found that patients with SSHL had higher NLR and PLR values, but both of these two inflammation markers had no significant correlation with the prognosis of sudden deafness. He believed that patients with higher NLR values responded better to anti-inflammatory drugs (steroids). Similarly, in 2020 Mehmet Eser Sancaktar (7) found that NLR and PLR decreased with the increase of the severity of hearing loss, and the patients with completely recovered and ascending audiogram had the highest values of NLR and PLRt, suggesting that high NLR and PLR may be prognostic factors of SSHL patients (21, 22). The heterogeneity of current findings on inflammation and SSHL may be related to the lack of classification for SSHL, as it is believed that the pathology of SSHL vary across clinical subtypes. Therefore, this study only selected patients with STD as research subjects to explore the correlation between inflammatory response and STD.

In this study, NLR has no significant correlation with the prognosis of STD, while PLR has a significant correlation with the efficacy of patients withSTD. The higher the PLR, the better the prognosis, which is consistent with the results of Mehmet Eser Sancaktar. Therefore, we speculated that inflammatory response played an important role in the occurrence of STD. Patients with elevated PLR have better response to anti-inflammatory drugs (steroids), so their prognosis are better. As a new peripheral inflammatory marker, PLR is associated with thrombosis and inflammation, which are closely related to the pathogenesis and progression of SSHL (23). In addition to its definite hemostatic and thrombotic functions, platelets are also considered to be an essential pro-inflammatory factor in atherosclerosis, allergy and rheumatoid arthritis. The increase of platelet count may promote platelet activation and increase the release of inflammatory mediators, resulting in an harmful inflammatory process to the body (24). While lymphocyte is considered to play an important role as immune regulation during all the inflammatory reaction stages of atherosclerosis (25), namely has a certain anti-inflammatory effect. So, PLR, which comes from the ratio of peripheral platelet count and lymphocyte count, includes the changes of platelets and lymphocytes, is more valuable than using any one of these two indicators in reflecting the inflammatoion level of our body. Therefore, the relationship between PLR and SSHL has gradually been concerned by more and more researchers.

The ROC analysis was finally applied in this study, and the effective rate of patients with age of ≤ 56 years and PLR value of >142.6 could reach to 78.1%, which was significantly higher than that of all the other three groups of patients. It has important clinical significance for guiding the prognosis of patients with STD in clinical practice.

## Limitation

This study did not monitor the peripheral inflammatory indexes at discharge, which is a defect of the study design. Therefore, it is impossible to indirectly show the correlation between various peripheral inflammatory markers and prognosis of SSHL. The role of inflammatory markers in the occurrence and development of sudden deafness needs to be confirmed by further well-designed randomized controlled studies.

## Conclusion

In summary, we found that Age and PLR are independent factors related to the prognosis of STD. The younger the onset age, the higher the PLR, the better the prognosis. The increase of PLR in patients with STD indicates that micro blood structure inflammation plays an important role in the occurrence of STD, suggesting that we need to re-explore the pathogenesis of STD and adjust the treatment to obtain better prognosis. In addition, PLR, which can be detected conveniently, combined with age, can predict the prognosis of patients with STD to a certain extent, the effective rate of patients with age of ≤ 56 years and PLR value of >142.6 could reach to 78.1%, which can help carry out personalized treatment reliably and economically.

## Data Availability Statement

The raw data supporting the conclusions of this article will be made available by the authors, without undue reservation.

## Ethics Statement

The studies involving human participants were reviewed and approved by the Peking University People's Hospital Ethical Permission Committee approved study (2021PHB149). The patients/participants provided their written informed consent to participate in this study. Written informed consent was obtained from the individual(s) for the publication of any potentially identifiable images or data included in this article.

## Author Contributions

XM contributed to the study conception and design and made critical revision for important intellectual content. TD supervised this research. TD, YK, JZ, and YJ contributed to the material preparation and data collection. TD, YK, and JZ contributed to the analysis and interpretation of data. TD and JZ wrote the first draft of the manuscript. All authors read and approved the final manuscript.

## Funding

This study was supported by Peking University People's Hospital Scientific Research Development Funds (RDL2021–14 and RDY2021–25), and National Key research and development program of China (2020YFC2005200).

## Conflict of Interest

The authors declare that the research was conducted in the absence of any commercial or financial relationships that could be construed as a potential conflict of interest.

## Publisher's Note

All claims expressed in this article are solely those of the authors and do not necessarily represent those of their affiliated organizations, or those of the publisher, the editors and the reviewers. Any product that may be evaluated in this article, or claim that may be made by its manufacturer, is not guaranteed or endorsed by the publisher.
